# Oxidative stress markers and septic acute kidney injury: Novel research avenue or road to nowhere?

**DOI:** 10.1186/s13613-016-0201-1

**Published:** 2016-10-10

**Authors:** Patrick M. Honore, Herbert D. Spapen

**Affiliations:** ICU Department, Universitair Ziekenhuis Brussel, Vrije Universiteit Brussel, 101, Laarbeeklaan, 1090 Jette, Brussels, Belgium

**Keywords:** Sepsis, Acute kidney injury, Oxidative stress, Erythrocyte superoxide dismutase, Biomarkers

In this issue of Annals of Intensive care, Costa et al. reported on the association between a lower erythrocyte superoxide dismutase (SOD)1 activity and the development of acute kidney injury (AKI) in septic shock patients. At a given cutoff level, SOD1 was found to predict sepsis-induced AKI (SAKI) earlier than the classic KDIGO criteria. The authors suggested that erythrocyte SOD1 activity could act as an early marker of SAKI and, by extension, as a novel target of SAKI biomarker research [1]. The study results certainly are original and innovative, but questions arise when considering their impact.

First, how strong is the oxidative stress response associated with SAKI? An honest answer is that we do not know! Sepsis indeed evolves along an inflammation–ischemia–reperfusion scenario. An important hallmark of the disease, amongst others, is a devastating oxidative burst substantiated by a harsh combat opposing tissue-aggressive reactive oxygen species (ROS) to intrinsic antioxidant body defense systems [2]. SAKI, however, is determined by a complex and highly treatment-dependent interplay between inflammation, microcirculatory dysfunction, bioenergetic failure, and tubular cell adaptation to injury [3]. Narrowing SAKI to a mere ischemia–reperfusion disorder is a too excessive simplification of its pathophysiology. Moreover, ischemia–reperfusion-induced oxidative stress is a generalized phenomenon, is difficult to quantify, and depends upon the duration of the ischemic insult and the speed of resuscitation both of which cannot be correctly estimated in the acute setting of sepsis. Erythrocyte SOD1 activity may thus be more an indicator of sepsis severity than of true kidney disease.

Second, is erythrocyte SOD1 an ideal biomarker? Arguably! Amidst the turmoil of oxidative aggression and antioxidant riposting, Costa et al. selected this particular scavenger probably because SOD is recognized as one of the first-line antioxidant “firefighters.” By definition, an ideal biomarker is released by injured organ-specific cells, is rapidly and repeatedly measurable, exhibits plasma concentrations proportional to the extent of injury, allows evaluating ongoing or receding organ damage, and reacts to therapy. SOD hardly corresponds to any of these criteria. Mammals possess three SOD isoforms: a predominantly intracellular copper–zinc SOD1, a mitochondrial manganese SOD2, and a copper–zinc SOD3 in the vascular extracellular space. Although catalyzing the same reaction, these isoforms are products of distinct genes and their specific subcellular location results in compartmentalized redox signaling. In fact, SOD2 might be a more specific marker than erythrocyte SOD1. SOD2 is abundantly present in the mitochondrial matrix where it is involved in dismutating superoxide anions generated by the respiratory enzyme chain [4]. Renal, and in particular proximal, tubular cells are extremely rich in mitochondria. The main pathophysiological phenotype of SAKI is tubular damage. Sepsis significantly cripples tubular cell integrity and function, which is reflected in the primarily tubuli-oriented quest for an adequate urinary SAKI biomarker [5, 6]. Changes in mitochondrial metabolism are increasingly thought to be a key issue for the progression of SAKI-related tubular damage. Dysfunctional mitochondria induce apoptosis and generate ROS, whilst remaining exposed to external ROS harassment in case of severe or prolonged oxidative stress [7]. SOD2 might also be more useful for follow-up because it has a plasma half-life of 5–6 hours compared with 6–10 minutes for SOD1 [8]. Finally, it is conceivable that other enzymatic players implicated in the anti-oxidative process (e.g., glutathione peroxidase and catalase) might equally well predict SAKI [9].

Thirdly, do the study results offer convincing evidence to pursue further research in this domain? Here, the answer is negative! An area under the receiver operating curve (AUROC) of 0.686 constitutes a rather weak correlation between SOD1 activity and SAKI development [1]. Such equally low specificity/sensitivity profile was the main reason why, for instance, the use of the Clinical Pulmonary Infection Score became heavily criticized for diagnosing ventilator-associated pneumonia [10]. To date, the urinary panel combining urinary insulin-like growth factor-binding protein 7 (IGFBP7) and tissue inhibitor of metalloproteinases-2 (TIMP-2) is considered to be the best performing biomarker set to predict the development of moderate or severe AKI. [TIMP-2]*[IGFBP7] AUROC curves for prediction and risk stratification of SAKI largely exceeded 0.80 in critically ill septic patients with either low or high non-renal Sequential Organ Failure Assessment scores [11].

Intensive care nephrologists crave to abandon serum creatinine as a late, highly biased, and poorly predictive marker of SAKI for an early, reliable, and kidney-specific biomarker. Within this context, Costa et al. provided interesting data of high scientific value. However, when “do not know,” “arguably” and “no” are the answers to pertinent questions regarding the clinical relevance of these findings, there is little reason to believe that a “novel avenue” of biomarker research in SAKI has been opened.

## Abbreviations

SOD: Superoxide dismutase
AKI: Acute kidney injury
SAKI: Sepsis-induced AKI
ROS: Reactive oxygen species
AUROC: Area under the receiver operating curve
TIMP-2: Tissue inhibitor of metalloproteinases-2
IGFBP7: Insulin-like growth factor-binding protein 7
